# Pathogenic drivers of lupus myocarditis and potential therapeutic targets

**DOI:** 10.1038/s12276-025-01580-4

**Published:** 2025-11-07

**Authors:** Yushan Liu, Katherine M. Murphy, Yu Fan Hung, Taejoon Won

**Affiliations:** Department of Pathobiology, College of Veterinary Medicine, University of Illinois Urbana-Champaign, Urbana, IL USA

**Keywords:** Autoimmunity, Systemic lupus erythematosus

## Abstract

Systemic lupus erythematosus (SLE) is a complex autoimmune disease that affects multiple organs simultaneously, complicating diagnosis and treatment. Despite extensive research, tissue-specific autoantigens and precise disease mechanisms remain unclear. Hallmark SLE autoantibodies primarily target nuclear antigens ubiquitously expressed across all tissues, limiting their diagnostic and therapeutic specificity. Myocarditis is a severe cardiovascular complication of SLE with a high mortality. However, not all patients with lupus myocarditis test positive for hallmark SLE antibodies, and their titers show no significant differences between patients with SLE with and without myocarditis, suggesting the involvement of additional, unidentified mechanisms. Autoimmunity against cardiac myosin heavy chain (MyHC) is a well-established driver of various forms of autoimmune myocarditis. However, the role of autoreactive T cells and autoantibodies targeting MyHC or other cardiac antigens in lupus myocarditis remains largely unknown. Here, in this Review, we offer an overview of the current knowledge on autoreactive T cells and autoantibodies identified in primary SLE or autoimmune myocarditis conditions from both clinical and preclinical studies. We also propose a novel two-stage model for lupus myocarditis pathogenesis, integrating both nuclear and cardiac antigen targets. Finally, we discuss antigen-specific regulatory T cells and chimeric antigen receptor T cells as promising therapeutic strategies for future research and clinical applications.

## New insights into lupus myocarditis and autoimmune mechanisms

Systemic lupus erythematosus (SLE) is a chronic autoimmune disease affecting multiple organs, including the heart. Lupus myocarditis, a severe heart inflammation, is a major complication of SLE. Researchers aim to understand the mechanisms behind lupus myocarditis. The authors explore how the immune system, particularly autoantibodies and autoreactive T cells, contributes to heart damage in SLE. They discuss how these immune components target both nuclear antigens and heart-specific proteins such as cardiac myosin heavy chain. The study highlights the role of tissue-resident memory T cells, which stay in the heart and may trigger inflammation during lupus flare-ups. The previous findings suggest that targeting specific immune pathways could lead to better treatments for lupus myocarditis. Future research may focus on developing therapies that regulate these immune responses, potentially improving outcomes for patients with SLE-related heart issues.

This summary was initially drafted using artificial intelligence, then revised and fact-checked by the author.

## Introduction

Systemic lupus erythematosus (SLE) is a chronic autoimmune disease that affects multiple organs, impacting approximately 3.41 million people worldwide^1^. SLE is characterized by autoantibody production and immune complex deposition, which may result in dysfunction or destruction of organs such as the joints, kidneys, lungs and heart^2^. Although the exact etiology of SLE is unclear, genetic predisposition and environmental factors are recognized as key contributors to its pathogenesis^2^. Cardiovascular involvement is one of the most common complications of SLE, being a major cause of morbidity and mortality^3^. In particular, lupus myocarditis is a severe manifestation, with a mortality rate of up to 23%^4^. Moreover, subclinical myocarditis has been identified in 30–60% of patients with SLE through autopsy studies, highlighting the significant risk of fatal cardiac events over their lifetime^5^. Despite its clinical importance, the underlying mechanisms driving lupus myocarditis remain poorly understood.

Autoantibodies are thought to play a significant role in lupus myocarditis, similar to their contribution to tissue damage in other organs, including the joints and kidneys, in SLE^6^. However, hallmark SLE autoantibodies primarily target nuclear antigens such as double-stranded DNA (dsDNA), Sjögren’s syndrome A (SSA), Sjögren’s Syndrome B (SSB), ribonucleoprotein (RNP) and Smith (Sm), which are ubiquitously expressed across all organs and cells rather than being specific to the heart^7^. T cells may also contribute to adaptive immunity underlying lupus myocarditis, as shown in endomyocardial biopsies (EMBs) from affected patients^8,9^. However, autoreactive T cells identified in patients with SLE so far, like autoantibodies, predominantly target nuclear proteins and lack tissue specificity^10–12^. This raises the question of whether non-tissue-specific autoantibodies or autoreactive T cells alone are sufficient to drive myocarditis development in SLE or whether other immunological factors are required.

In innate immunity, type I interferons (IFN-I) are well-established cytokines involved in SLE pathogenesis. It is known that nucleic-acid-containing immune complexes bind to endosomal Toll-like receptors (TLRs), followed by the activation of downstream signaling pathways and the production of IFN-ɑ, a subtype of IFN-I, in both plasmacytoid dendritic cells and non-hematopoietic cells in patients with SLE^13^. IFN-I is believed to contribute to further inflammation and tissue damage^13^. In addition, the deficiency of complement proteins, another component of innate immunity, is associated with increased susceptibility to SLE, as impaired clearance of apoptotic cells and autoantigens promotes autoantibody production and immune complex formation^14^. Even in patients with SLE without complement deficiencies, low levels of complement C3 or C4 are often observed due to their consumption during active inflammation requiring immune complex formation^14^. However, no studies so far have demonstrated lupus myocarditis specifically associated with IFN-I or complement in SLE.

The cardiac myosin heavy chain (MyHC), encoded by the *MYH6* gene, is a well-known heart-specific antigen and can be targeted by both autoreactive T cells and autoantibodies in various forms of myocarditis^15,16^. Recent studies have revealed that hyperactivation of MyHC-specific T cells, caused by blocking the programmed cell death protein 1 (PD-1)/programmed death-ligand 1 (PD-L1) pathway, contributes to the onset and progression of immune checkpoint inhibitor (ICI)-associated myocarditis in both patients and murine models^17,18^. In addition to MyHC, several other heart-specific proteins such as adenine nucleotide translocator 1 (ANT1), β1-adrenergic receptor (β1AR), cardiac troponin I (cTnI), muscarinic M2 receptor (M2R) and sarcoplasmic/endoplasmic reticulum Ca^2+^ adenosine triphosphatase 2a (SERCA2a) have been proposed as autoantigens that can activate cellular and humoral immunity, leading to myocarditis development^19,20^. Interestingly, some SLE mouse models that developed myocarditis exhibited elevated anti-MyHC antibody titers^21–24^. This may suggest a potential role of autoimmunity against heart-specific antigens in lupus myocarditis, although there are limited studies.

In this Review, we highlight key drivers in the pathogenesis of lupus myocarditis, with a focus on the adaptive immune system. We provide the latest insights into autoantibodies and autoreactive T cells associated with primary SLE and autoimmune myocarditis across clinical and preclinical studies. Furthermore, we propose a potential mechanism underlying lupus myocarditis and discuss antigen-specific regulatory T (T_reg_) cells as a potential targeted therapeutic strategy.

## Challenges in the diagnosis of lupus myocarditis

The clinical manifestations of lupus myocarditis often overlap with those of viral myocarditis, with common symptoms including chest pain, arrhythmia and palpitations, complicating differential diagnosis^25^. Although EMB is the diagnostic gold standard, its invasive nature and associated procedural risks limit its use in routine clinical practice^26^. To address this, noninvasive diagnostic approaches, such as cardiac imaging, serologic immune markers and circulating biomarkers, are currently used, clinically tested or under development^5^.

Echocardiography can detect structural and functional abnormalities, including arrhythmias, myocardial infarction and conduction disturbances^27,28^. However, its findings are generally nonspecific and insufficient for a definitive diagnosis of lupus myocarditis without supportive evidence^29,30^. Advanced approaches such as cardiac magnetic resonance imaging (CMRI) and positron emission tomography provide more sensitive and detailed assessments, yet their application in lupus myocarditis remains constrained^29,30^. Many patients suspected of lupus myocarditis also present with concomitant lupus nephritis, where renal impairment precludes the safe use of contrast agents^31,32^. Furthermore, patients with severe cardiac dysfunction may be unable to perform the breath-holding maneuvers required for CMRI scanning^33^.

Serologic immune markers may provide complementary diagnostic support in lupus myocarditis. Notably, it has been reported that anti-SSA antibodies are detected at a higher prevalence in patients with lupus myocarditis than in those with SLE without cardiac involvement^34^. Similarly, cardiac injury biomarkers such as troponin and creatine kinase may be elevated during lupus myocarditis, although both markers lack specificity and must be cautiously interpreted in conjunction with other clinical and imaging findings^31–34^. Thus, these challenges highlight the need to better understand the cellular and molecular mechanisms underlying lupus myocarditis to facilitate the development of safer, more reliable and clinically convenient diagnostic strategies, as well as targeted therapeutic and preventive approaches.

## Contribution of autoantibodies to lupus myocarditis pathogenesis

### Hallmark SLE autoantibodies targeting nuclear antigens

**Fig. 1:**
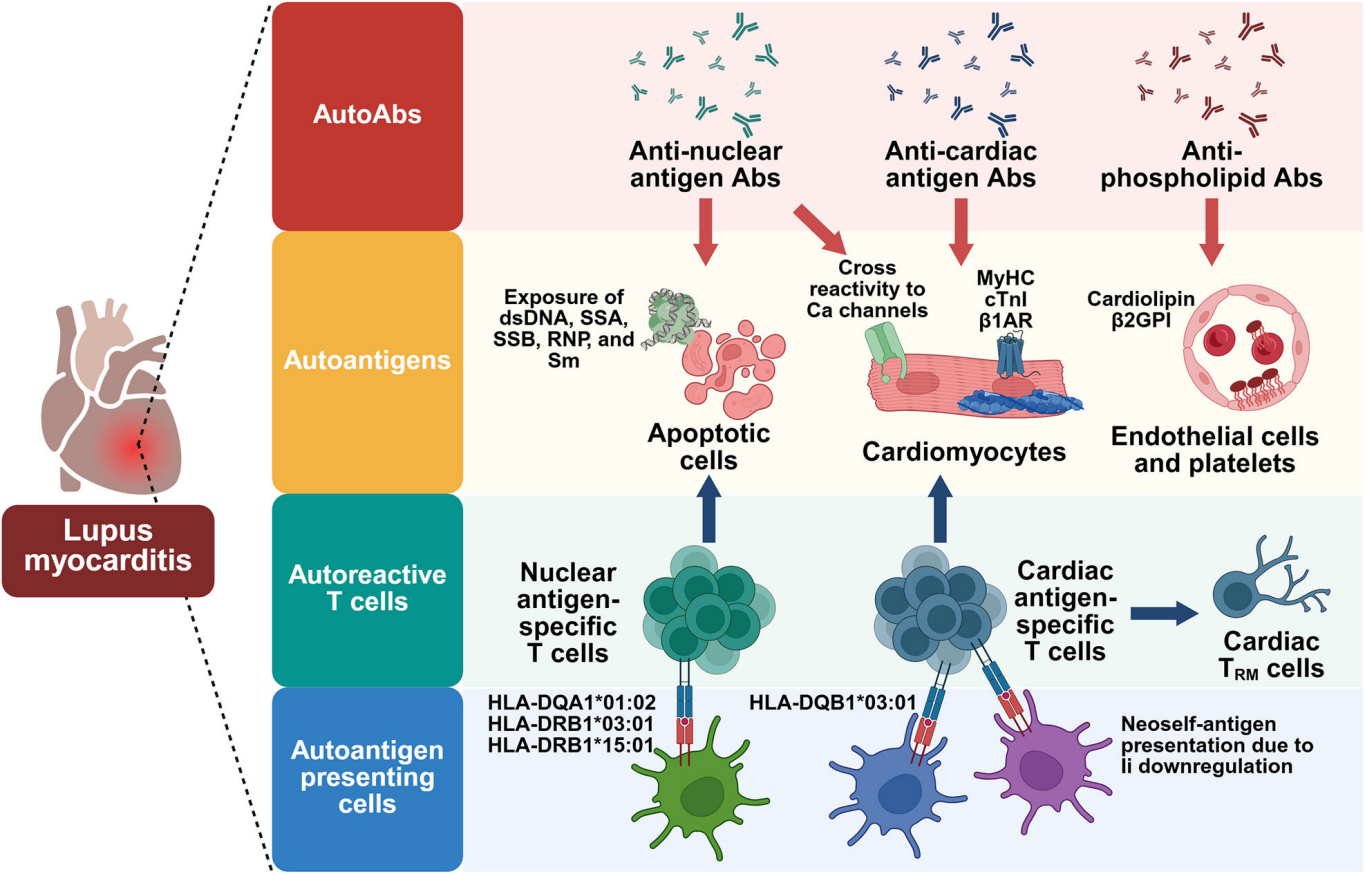
Key players in the pathogenesis of lupus myocarditis. The activation of antibodies (Abs) and T cells targeting nuclear antigens such as double-stranded DNA (dsDNA), Sjögren’s syndrome A (SSA), Sjögren’s syndrome B (SSB), ribonucleoprotein (RNP) and Smith (Sm) is initiated by apoptotic cells, contributing to the development of lupus myocarditis. In addition, cardiac antigen-specific autoantibodies and autoreactive T cells, targeting cardiac MyHC, cardiac troponin I (cTnI) and β1-adrenergic receptor (β1AR), play a pathogenic role by damaging cardiomyocytes. MyHC-specific T cells can further differentiate into tissue-resident memory T (T_RM_ cells in the heart. Some anti-nuclear antigen antibodies may recognize cardiomyocytes as an antigen through cross-reactivity to calcium (Ca) channels. The development of autoreactive T cells is associated with the expression of susceptible human leukocyte antigen (HLA) alleles and the presentation of neoself-antigens due to loss or decrease of invariant chain (Ii) expression. Furthermore, antiphospholipid antibodies bind directly to cardiolipin and β2 glycoprotein I (β2GPI) on endothelial cells and platelets, driving cardiovascular inflammation.

Autoantibodies against nuclear antigens such as dsDNA, SSA, SSB, RNP and Sm are commonly detected in patients with SLE, although disease manifestation, severity and progression are widely heterogeneous^7^ (Fig. 1). In lupus myocarditis cases, clinical studies have shown that anti-dsDNA antibodies are present in over 70% of patients, while the positivity for anti-SSA, anti-RNP and anti-Sm antibodies widely varies between 20% and 70%^35–38^. By contrast, anti-SSB antibodies tested positive in relatively fewer patients (less than 23%) compared with other antibodies^35–38^. Among these autoantibodies, the cardiotoxicity of anti-SSA autoantibodies has been recognized. The titer of anti-SSA antibodies in patients with lupus myocarditis was a significant predictor for fibrosis and necrosis in the heart, confirmed by late gadolinium enhancement on CMRI, with high sensitivity and specificity^38^. Patients who tested positive for anti-SSA antibodies exhibited a significantly higher prevalence of myocarditis and conduction defects than those who tested negative^34^. Beyond myocarditis, a large population-based study showed that anti-SSA and anti-SSB seropositivity is associated with an increased risk of ischemic heart disease, particularly in patients under 40 years^39^. In addition, the occurrence of arrhythmia was strictly associated with anti-SSA autoantibody levels in adult patients with connective tissue disease (CTD)^40^. In neonatal cases, multiple studies have reported that anti-SSA autoantibodies can be passively transferred from pregnant mothers to fetuses, accumulate in fetal cardiac tissue and cause lethal congenital heart block^41,42^.

As a potential mechanism, anti-SSA antibodies are known to recognize the Ro52 protein on the surface of apoptotic cells, forming immune complexes that stimulate IFN-I production^43^. In addition, anti-SSA and anti-SSB autoantibodies promoted the production of tumor necrosis factor (TNF) in macrophages, eliciting inflammation^44^. However, because nuclear antigens are ubiquitously expressed across all organs, it is still unclear how autoantibodies against them can contribute to specifically myocarditis and other cardiac complications in patients with SLE. As a clue, it has been reported that anti-SSA and anti-SSB autoantibodies can cross-react with cardiac L-type and T-type calcium channels, inhibiting calcium current and disrupting calcium homeostasis in the heart^41,45^ (Fig. 1). Despite the cross-reactivity, previous studies have shown that serum levels of anti-SSA and anti-SSB autoantibodies were comparable between patients with SLE with or without myocarditis^36–38^. This suggests that additional mechanisms may drive the accumulation and pathogenic activity of anti-nuclear antigen autoantibodies, warranting further investigation in future research.

### Autoantibodies against phospholipid

Antiphospholipid autoantibodies are the hallmark of antiphospholipid syndrome (APS), which is an autoimmune thrombo-inflammatory disease frequently complicated in patients with SLE^46^. Among these antibodies, anti-cardiolipin and anti-β2 glycoprotein I (β2GPI) antibodies as well as lupus anticoagulant are known to be detected in approximately 50% or fewer patients with lupus myocarditis, suggesting their pathogenic role^35–38^ (Fig. 1). Some studies showed that the level of antiphospholipid autoantibodies is comparable between patients with SLE with or without myocarditis^37,38^. However, a study by Ramirez et al. revealed that the presence of anti-β2GPI antibodies was more frequent in patients with SLE with myocarditis compared with those without myocarditis and that anti-β2GPI positivity was significantly correlated with lupus myocarditis onset^36^. Interestingly, in this study, the authors speculated that the increase of anti-β2GPI antibodies in patients with lupus myocarditis is due to a history of APS, suggesting the involvement of cardiovascular complications of APS^36^. A preclinical study reported that B6.*sle1.sle2.sle3* triple congenic mice, a well-established SLE model, develop myocarditis when treated with resiquimod, a TLR7/8 agonist^47^. This model also showed elevated levels of autoantibodies targeting cardiolipin and β2GPI, suggesting their roles in lupus myocarditis pathogenesis (Table 1). Beyond myocarditis, the presence of lupus anticoagulant in SLE patients was significantly associated with myocardial fibrosis, as detected by CMRI^48^. Another study demonstrated that increased anti-cardiolipin autoantibody level is strongly associated with other cardiac complications such as valvular lesions, pericardial involvement and myocardial dysfunction in patients with SLE^49^. It is also reported that the positivity of antiphospholipid antibodies in early SLE is associated with subsequent vascular events such as venous thrombosis, pulmonary embolism, coronary disease and cerebrovascular attack^50^.

**Table 1:** Murine models for studying lupus myocarditis.

Mouse strain	Genetic manipulation or pharmacological treatment	Organs undergoing autoimmune inflammation	Identified targets of autoantibodies	Reference
MRL.*lpr*	*CD274*^−/− (PD-L1 KO)^	Heart and lungs	Cardiac myosin and cardiac troponin I	^21^
MRL	*Pdcd1*^−/− (PD-1 KO)^	Heart, salivary glands, stomach, lungs and liver	Cardiac myosin	^22^
C57BL/6	*Trex1*^−/−^	Heart, skeletal muscle, tongue, skin, stomach and brain	Cardiac myosin and junctophilin-2	^23^
Triple cross between CFN	Resiquimod treatment	Heart	Cardiac myosin and cardiac troponin I	^24^
B6.*Sle1.Sle2.Sle3* triple congenic	Resiquimod treatment	Heart	dsDNA, β2GPI and cardiolipin	^47^
NZM2410	–	Heart and kidneys	–	^141^

It is well known that patients with primary APS experience cardiac complications such as valvular disease and acute myocardial infarction^51^. Mechanistically, in patients with APS, anti-β2GPI autoantibodies create immune complexes associated with thrombotic events and also directly bind to endothelial cells and platelets, followed by the initiation of the coagulation cascade^46^. Furthermore, these autoantibodies can activate the innate immune system such as complement, monocytes and neutrophils, indicating their potential contribution to inflammation beyond thrombosis in the heart and vasculature^46^. Indeed, several case reports have shown the development of acute myocarditis in patients with APS positive for antiphospholipid antibodies, further supporting their potential pathogenic role in lupus myocarditis^52,53^.

### Autoantibodies specific for cardiac antigens

Autoantibodies targeting heart-specific antigens such as MyHC, cTnI and β1AR have long been studied to understand the pathogenesis of autoimmune myocarditis, not limited to SLE conditions^19^ (Fig. 1). Anti-MyHC autoantibodies are detected in the serum of patients with various cardiac inflammatory conditions, including autoimmune myocarditis, viral myocarditis and myocardial infarction^54–56^. In addition, these antibodies are detected in 42–52% of EMB samples from patients with myocarditis, supporting their pathogenic role in the heart^57^. However, because anti-cardiac antigen antibodies are not routinely tested in patients with SLE, there is limited clinical evidence connecting these autoantibodies to lupus myocarditis. A case report showed that anti-MyHC antibodies were detected in patients with SLE with constrictive pericarditis, but no studies so far have shown their presence in lupus myocarditis^58^. Despite the lack of clinical studies, several animal models support a link between anti-MyHC autoantibodies and lupus myocarditis (Table 1). MRL and MRL.*lpr* mice, well-established SLE mouse models, spontaneously developed autoimmune myocarditis with a high titer of anti-MyHC antibodies when the PD-1/PD-L1 pathway was genetically blocked^21,22^. Similarly, *Trex1*-deficient mice or C57BL/6, FVB and NOD (CFN) mice (a triple cross between C57BL/6, FVB and NOD strains), both models for SLE and other autoimmune diseases, developed myocarditis along with the presence of MyHC-specific autoantibodies^23,24^. These findings highlight the need for further research into the role of anti-MyHC autoantibodies in lupus myocarditis pathogenesis.

A recent study using an advanced proteomics approach identified novel heart-specific antigen candidates potentially targeted by autoantibodies in lupus myocarditis: disco-interacting protein 2 homolog A (*DIP2A*), LIM domain 7 (*LMO7*), poliovirus receptor (*PVR*) and plasminogen activator, urokinase receptor (*PLAUR*)^59^. In this study, engineered human cardiac tissues were developed using human induced pluripotent stem cells to assess the cardiotoxicity of immunoglobulin G fractions from patients with lupus myocarditis^59^. The study showed direct binding of patient immunoglobulin G to stressed cardiac tissues, leading to impaired calcium handling and mitochondrial function in cardiomyocytes as well as increased proliferation of fibroblasts. These findings strongly suggest the pathogenic role of heart-specific autoantibodies in lupus myocarditis pathogenesis and highlight the potential of advanced techniques for identifying antigen targets.

## Pathogenic role of T cells in lupus myocarditis

### Autoreactive T cells targeting nucleoproteins

T cells play a crucial role in the pathogenesis of SLE by producing inflammatory cytokines, stimulating autoantibody secretion in B cells and contributing to autoimmune memory^60^. Studies have demonstrated that T cells in patients with SLE are chronically activated due to altered epigenetic regulation, oxidative stress and metabolic dysfunction^61,62^. Under SLE conditions, the expression of T cell receptor subunit cluster of differentiation 3ζ (CD3ζ) is downregulated, while the Fcε receptor Iγ (FcεRIγ) chain is upregulated, leading to inappropriate hyperactivation of downstream signaling pathways and aberrant T cell stimulation^63^. Mechanistically, DNA hypomethylation, lipid raft abnormally enriched with gangliosides, mammalian target of rapamycin (mTOR) pathway activation and elevated glycolysis are known to contribute to T cell hyperactivation in SLE^64–67^.

Similar to hallmark autoantibodies, U1-RNP70 and other nucleoproteins have been studied as autoantigens targeted by T cells in SLE at both clinical and preclinical levels. Clinical studies have reported that nuclear antigens such as U1-RNP70, SmD1, histones, SSA and SSB are recognized by CD4^+^ T cells in patients with SLE, resulting in cytokine production including interleukin (IL)-17, IL-10 and IFN-γ^10–12^ (Fig. 1). These T cells are detectable not only in peripheral blood mononuclear cells but also in urine, suggesting their potential as novel biomarkers^10^. The significance of autoreactive T cells specific for these nuclear proteins in SLE has been confirmed in animal studies. In murine SLE models, including NZB×NZW F1 and MRL.*lpr* mice, unprimed CD4^+^ T cells were activated in response to U1-RNP70 peptides, leading to autoantibody production in B cells against the same antigen^12,68^. In addition, histone proteins and SSB were recognized by autoreactive T cells in SWR×NZW F1 lupus mice and in vitro murine T cell stimulation assays, respectively^69,70^. Besides nuclear proteins, vimentin and annexin 2 have been identified as targets of autoreactive CD4^+^ T cells in patients with lupus nephritis^71^.

U1-RNP70 is a well-established nucleoprotein and known as a primary target of hallmark autoantibodies in mixed CTD (MCTD), a rheumatological disease that primarily affects the skin and muscles, and largely overlaps symptoms and pathophysiology with SLE^72,73^. Similar to SLE, autoreactive T cells targeting the U1-RNP70 antigen have been identified in patients with MCTD^12,74^. Notably, myocarditis has been known as one of the cardiovascular complications in MCTD, evidenced by case reports^75,76^. This suggests that U1-RNP70-specific autoreactive T cells may be a key driver in the onset and progression of myocarditis due to both SLE and MCTD conditions. However, U1-RNP70 is one of the ubiquitous nuclear proteins across all different tissues, and the mechanisms underlying its heart specificity remain unclear.

### Cardiac antigen-specific T_RM_ cells

MyHC represents a critical self-antigen involved in autoimmune myocarditis (Fig. 1). The *MYH6* gene encoding MyHC is notably absent in medullary thymic epithelial cells, which are essential for the thymic negative selection of T cells recognizing tissue-restricted self-antigens^15^. Consequently, MyHC-specific T cell clones can avoid negative selection and migrate to the periphery, potentially becoming cardiac-specific autoreactive T cells^15^. The presence and pathogenicity of MyHC-specific autoreactive T cells have been studied in various inflammatory cardiac conditions, including viral myocarditis, autoimmune myocarditis, ICI-associated myocarditis and dilated cardiomyopathy^15,17,77^. Supporting these findings, extensive research in murine models has consistently confirmed autoreactive T cells targeting MyHC as critical players in autoimmune myocarditis^15,17,18,78–80^. Despite accumulating evidence emphasizing the pathogenic role of MyHC-specific autoreactive T cells, their involvement and underlying mechanisms in lupus-associated myocarditis remain largely understudied, highlighting a significant gap for future research efforts. Previous animal studies using lupus-prone mice showed that pathogenic T cells drive spontaneous myocarditis development in MRL, MRL.*lpr* and *Trex1*-deficient mice, as well as resiquimod-induced autoimmune myocarditis in CFN mice^21–24^. Although the antigenic target of these T cells has not been identified, humoral autoimmunity against MyHC was confirmed by antibody profiling in their studies^21–24^ (Table 1). These findings suggest the involvement of MyHC-targeting autoreactive T cells in lupus myocarditis pathogenesis as well as autoantibodies. Moreover, clinical and animal studies identified that ANT_1_, β1AR, SERCA2a and cTnI can be antigenic targets for T cells in autoimmune myocarditis^19^.

Tissue-resident memory T (T_RM_) cells are a subset of memory T cells that reside in peripheral tissues without recirculation and provide immediate immune responses upon re-exposure to antigens^81^. Clinical studies have revealed the significance of CD8^+^ or CD4^+^ T_RM_ cells in various autoimmune diseases, including Crohn’s disease, rheumatoid arthritis, encephalomyelitis and cutaneous lupus erythematosus^82–85^. Several studies identified CD8^+^ T_RM_ cells in the kidneys of patients and mice with lupus nephritis, suggesting the potential role of T_RM_ cells in SLE pathogenesis^86–88^. Remarkably, a recent study discovered heart-resident T_RM_ cells in the pericardial effusion of patients with ischemic or dilated cardiomyopathy, characterized by CD69, PD-1 and CXC chemokine receptor 6 (CXCR6) expression^89^. This study also shows through mouse experiments that a previous history of subclinical cardiac inflammation or ischemic injury can induce the recruitment and expansion of cardiac T_RM_ cells, ultimately resulting in active myocarditis when immune tolerance was later disrupted by ICI treatment^89^. Furthermore, MyHC-specific autoreactive T cells were one of the subsets in these cardiac T_RM_ cells^89^. These findings suggest that primary adverse cardiac events, even if mild or subclinical, can contribute to the development of pathogenic cardiac T_RM_ cells as a risk factor and that a subsequent wave of autoimmunity or immune dysregulation can then activate these T cells, driving the progression of active myocarditis. Although the specific role of MyHC-targeting T cells or cardiac T_RM_ cells in lupus myocarditis remains unknown, SLE conditions may contribute to either or both the initial cardiac damage that recruits T_RM_ cells and later immune-related events that activate them. Future research should focus on elucidating the precise mechanisms by which heart-targeting T_RM_ cells contribute to cardiac manifestations in SLE.

### MHC and autoantigen presentation

The major histocompatibility complex (MHC), also known as human leukocyte antigen (HLA), is expressed on the cell surface to present self or foreign antigens to T cells. A strong association between HLA class II alleles and autoimmune disease has been extensively studied, suggesting activated autoreactive T cells and compromised T_reg_ cells as potential mechanisms^90^ (Fig. 1). The HLA-DQA1*01:02, HLA-DRB1*03:01 and HLA-DRB1*15:01 alleles have been linked to an increased risk or prevalence of SLE across diverse racial groups^91–93^. In heart diseases, the HLA-DQB1*03:01 allele has been reported at a higher frequency in patients with idiopathic dilated cardiomyopathy, as evidenced by the development of spontaneous myocarditis in transgenic mice expressing this allele^94–96^. These studies identified the presence of autoantibodies and autoreactive T cells against MyHC in mice with myocarditis^94,95^. Interestingly, in patients with SLE, the HLA-DQB1*03:01 allele has also been associated with lupus anticoagulant positivity, a hallmark antibody of APS^97^. Another study reported a link between lupus anticoagulant and myocardial fibrosis, a potential consequence of myocarditis, in patients with SLE^48^. These findings suggest a possible pathogenic connection between specific HLA haplotypes, autoantibodies and myocarditis in patients with SLE.

A recent study by Mori et al. has proposed a novel mechanism for SLE pathogenesis, suggesting that the absence or downregulation of the invariant chain (Ii) enables MHC class II to present neoself-antigens to autoreactive T cells, contributing to disease development^98^ (Fig. 1). Under normal conditions, Ii prevents the binding of unwanted peptides and unfolded proteins to the groove of MHC class II molecules and drives the MHC class II into the endosomal–lysosomal pathway, where antigen peptide binding occurs^99^. Based on this mechanism, neoself-antigens that can activate heart-targeting autoreactive T cells in patients with SLE with impaired Ii chain may become a critical driver in the onset and progression of lupus myocarditis.

## Potential mechanisms underlying lupus myocarditis

**Fig. 2:**
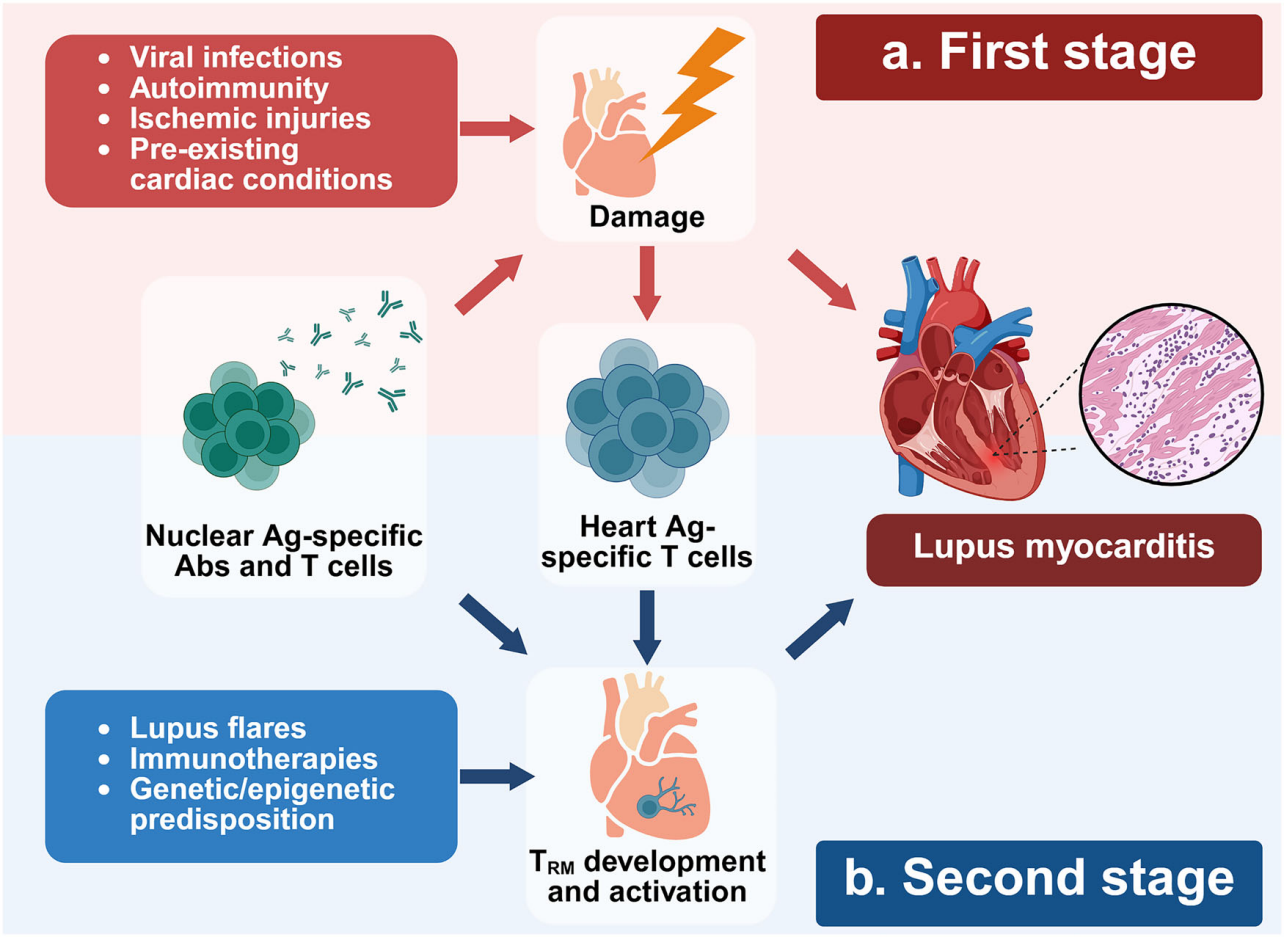
Proposed two-stage pathogenesis model for lupus myocarditis. **a** In the initial stage, adverse events such as viral infections, autoimmunity, ischemic injuries and pre-existing cardiac conditions cause primary heart damage. This damage exposes nuclear antigens (Ag), activating lupus hallmark antibodies (Abs) and autoreactive T cells, which contribute to lupus myocarditis development. Even in individuals who do not progress to myocarditis, cardiac antigen-specific autoreactive T cells can still be recruited and expanded. Some of these cells reside in the heart as T_RM_ cells. **b** In the second stage, immune dysregulation such as lupus flares, immunotherapies and genetic/epigenetic predisposition triggers cardiac T_RM_ cell activation, further driving lupus myocarditis. Lupus hallmark autoAbs and autoreactive T cells exacerbate myocarditis.

Based on current knowledge, we propose a two-stage model for the mechanisms driving lupus myocarditis (Fig. 2). In the first stage, initial heart damage arising from viral infections, autoimmunity, ischemic injuries or pre-existing cardiac conditions leads to cell death and exposure of nuclear antigens (Fig. 2a). In some patients with SLE, this damage directly activates nuclear antigen-targeting antibodies and T cells, resulting in lupus myocarditis development. Specifically in the context of autoimmunity at this stage, the cross-reactivity of anti-SSA antibodies with cardiac antigens or the binding of antiphospholipid antibodies to cardiac endothelial cells may directly induce apoptosis of heart-resident cells or cause tissue damage through immune complex formation^41,45,46^. It is known that primary myocarditis, typically caused by viral infections, is often subclinical and self-limiting in healthy individuals^100^. Similarly, some patients with SLE may not progress to active myocarditis or may experience only mild disease due to limited initial damage at the first stage. However, in these patients, autoreactive T cells specifically targeting cardiac antigens may still be recruited to the heart and persist as T_RM_ cells^89^. Particularly, MyHC-specific autoreactive T cells can be developed and recruited following initial cardiac injury, as these T cells bypass the thymic negative selection^15^.

In the second stage, systemic immune dysregulation, triggered by lupus flares, immunotherapies and genetic/epigenetic predisposition, can hyperactivate cardiac T_RM_ cells in patients with SLE, leading to active myocarditis (Fig. 2b). T_RM_ cells expressing CD69 and/or CD103 have been identified in the kidneys of patients and mice with lupus nephritis, suggesting their presence and pathogenic role in the heart under SLE conditions^86–88^. The PD-1/PD-L1 pathway is a key immune tolerance mechanism to restrict cardiac T_RM_ cell activation^89^. PD-1 inhibitor treatment, a cancer immunotherapy, markedly increased the frequency and severity of myocarditis in mice enriched with cardiac T_RM_ cells compared with mice with no enrichment^89^. This is supported by a clinical report of myocarditis in a patient with SLE following ICI therapy^101^. Interestingly, genetic studies revealed that the PD1.3 polymorphism has been linked to increased SLE susceptibility, whereas PD1.6 is associated with decreased risk, indicating the contribution of PD-1 variants to loss of T_RM_ regulation and lupus myocarditis pathogenesis^102,103^. In addition, abnormal activation of mTOR signaling in SLE may predispose patients to lupus myocarditis by promoting the development of cardiac antigen-specific T_RM_ cells^104^. A study revealed that inhibition of mTOR signaling limits T_RM_ cell formation by reducing effector T cell accumulation in the periphery^105^. Beyond cardiac T_RM_ cells, autoantibodies and autoreactive T cells specific for nuclear antigens can further amplify inflammation in the heart through epitope spreading at the second stage (Fig. 2b). New antigens, most likely nuclear proteins, released from damaged cardiac cells at the first or second stage generate additional autoreactive B and T cell responses, exacerbating lupus myocarditis^106,107^.

In additoin, T cells recognizing neoself-antigens in patients with Ii chain downregulation are expected to play a critical role in lupus myocarditis development at both stages^98^. In the first stage, these T cells can directly damage cardiac tissue through recognizing cardiac-specific neoself-antigens, leading to the exposure of nuclear antigens. After priming, they may persist as inactive T_RM_ cells in the heart. Upon systemic flare conditions at the second stage, they can become highly activated, contributing to lupus myocarditis pathogenesis.

## Protective role of T_reg_ cells in lupus myocarditis

### Imbalance between regulatory and effector T cells

T_reg_ cells are essential for maintaining immune homeostasis, and an imbalance between T_reg_ cells and effector T (T_eff_) cells contributes to autoimmune disease pathogenesis^108^. In patients with SLE, reduced frequency and function of T_reg_ cells have been observed, while pathogenic T_eff_ cells and their cytokine production were increased^109–111^. Metabolic dysregulation is suggested as a key factor driving T cell imbalance in SLE. It is known that the mTOR signaling pathway downregulates immunosuppressive function and forkhead box P3 (FoxP3) expression in T_reg_ cells, indicating an overactivated mTOR signaling pathway in SLE conditions as a mechanism underlying reduced T_reg_ cell development and function^112^. By contrast, mTOR signaling pathway activation is strongly associated with the hyperactivation of pathogenic T cells in SLE^66^. A clinical study showed that IL-21 produced by activated T_eff_ cells, specifically helper T (T_H_) 17 cells, further compromises T_reg_ cell development and function in patients with SLE^113^.

The balance between T_reg_ and T_eff_ cells is also important in cardiovascular health. A clinical study showed that patients with chronic heart failure had a reduced number of Tregs that did not recover even after 6 months of cardiac resynchronization therapy^114^. These patients exhibited persistent inflammatory conditions characterized by increased cytotoxic T lymphocytes despite therapy^114^. Similarly, patients with autoimmune myocarditis and its sequela, dilated cardiomyopathy, showed reduced T_reg_ cells, along with elevated T_H_1 and T_H_17 cell populations and their inflammatory cytokine production^77,115^. The imbalance between T_reg_ and T_H_17 cells in both viral myocarditis and autoimmune myocarditis was further confirmed in mouse models^116,117^. One of these studies using single-cell ribonucleic acid (RNA) sequencing analysis revealed the predominance of T_H_17 cells and cytotoxic T lymphocytes in the heart with myocarditis, despite the presence of T_reg_ cells^117^. Moreover, adoptive transfer of CD4^+^ T cell populations with T_reg_ cell depletion induced fatal myocarditis and multiorgan autoimmune inflammation in BALB/c nude mice^118^. In SLE conditions, impaired T_reg_ cell generation and immunoregulatory function, coupled with increased Th17 cell differentiation and expansion, may create a highly proinflammatory environment that promotes myocarditis development.

### Therapeutic potential of T_reg_ cells

T_reg_ cell supplementation has been investigated as a potential cell-based immunotherapy for autoimmune diseases, which are characterized by a reduced number and impaired function of T_reg_ cells^108^. The transfer of autologous polyclonal T_reg_ cells to patients with various autoimmune conditions has shown benefits, including slowed disease progression. In the clinical trial NCT03241784, patients with amyotrophic lateral sclerosis who received autologous ex vivo expanded T_reg_ cells exhibited reduced clinical progression with increased circulating T_reg_ cell number and immunosuppressive function^119^. Similarly, after adoptive transfer of T_reg_ cells to a patient with ulcerative colitis, the clinical activity disease score decreased from 7 to 2, along with enriched T_reg_ cells in the gut^120^. T_reg_ cell therapy has also been widely tested in preclinical animal models of both SLE and cardiovascular diseases. In lupus-prone NZB×NZW F1 mice, the adoptive transfer of ex vivo expanded T_reg_ cells improved renal disease, proteinuria and mortality rate, possibly by restoring the balance between T_reg_ and T_eff_ cells^121,122^. Furthermore, T_reg_ cell therapy protected mice from cardiovascular diseases such as hypertension, vascular inflammation, atherosclerosis and viral myocarditis through the reduction of heart antigen-specific autoimmunity, the resolution of inflammation involving both innate and adaptive immunity and the restoration of cardiovascular function^123–125^. These findings suggest that adoptive transfer of T_reg_ cells may serve as a potential therapeutic strategy for lupus myocarditis by reestablishing immune balance and mitigating inflammation.

## Antigen-specific T_reg_ cells as a potential cell-based immunotherapy for lupus myocarditis

### Antigen-specific T_reg_ cells

Beyond the use of autologous T_reg_ cells, antigen-specific T_reg_ cell therapy has emerged as a targeted approach for autoimmune diseases. Single antigen-specific T_reg_ cells might exhibit improved immunosuppression and tissue protection compared with polyclonal T_reg_ cells due to precise organ targeting, increased antigen specificity and reduced off-target effects^126^. It is reported that more than 25 clinical trials have been conducted to explore the benefits of antigen-specific T_reg_ cell therapy in treating various immune-related diseases such as type 1 diabetes, Crohn’s disease and graft-versus-host disease^126^. In the Crohn’s and T_reg_ Cells Study (CATS1) clinical trial, researchers investigated the efficacy of ovalbumin-specific T_reg_ cells in patients with refractory Crohn’s disease^127^. Six to eight weeks after transfer of antigen-specific T_reg_ cells, 40% of patients showed clinical improvement with a reduction in inflammatory monocytes^127^. Notably, for lupus nephritis treatment, engineered human T_reg_ cells that can specifically target the Sm antigen, a nucleoprotein involved in SLE, have been generated and shown enhanced antigen specificity and immunosuppressive activity compared with polyclonal T_reg_ cells, as well as their therapeutic potential in a humanized mouse SLE model^128^. Although no studies have yet reported antigen-specific T_reg_ cell therapy for heart diseases, cardiac antigen-specific T_reg_ cells may be a promising candidate for a targeted cell-based therapeutic approach for various inflammatory cardiac conditions, including lupus myocarditis.

### CAR-T cell and CAR-T_reg_ cell therapies

Chimeric antigen receptor (CAR)-T cells are engineered T cells equipped with an antigen receptor composed of a single-chain variable fragment that efficiently recognizes a specific extracellular antigen, triggering downstream intracellular signaling pathways for further T cell activation^129^. CAR-T cells were originally developed to target tumor antigens for cancer treatment and approved by the Food and Drug Administration, but they are currently being adapted for autoimmune disease treatment^126,130^. In the context of SLE, CD19 is a promising target of CAR-T cell therapy because it is selectively expressed on B cells and plasmablasts, the primary producers of anti-nuclear antigen and anti-dsDNA autoantibodies. Preclinical studies have exhibited that anti-CD19 CAR-T cells effectively deplete B cells in lupus-prone mice, leading to improved lupus nephritis^131,132^ (Table 2). Clinically, anti-CD19 CAR-T cell therapy combined with lymphodepletion with fludarabine and cyclophosphamide has successfully improved clinical symptoms in patients with SLE by resetting the immune system and normalizing lupus-related parameters^133^. No significant adverse effects on major organs have been observed between 4 and 29 months after this therapy, although the patient sample size has been limited^134^. Nevertheless, the anti-CD19 CAR-T cell approach may only be effective for SLE driven primarily by B cells and plasmablasts, whereas its therapeutic benefit is limited in autoimmune conditions mediated by plasma cells or T cells, both of which lack CD19 expression. Furthermore, an increased risk of infections may be a limitation of CAR-T cell therapy.

**Table 2:** Comparison between different CAR-T cell therapies for lupus myocarditis treatment.

	Anti-CD19 CAR-T cells	Anti-CD19 CAR-T_reg_ cells	Cardiac antigen-specific CAR-T_reg_ cells
Target antigen	CD19	CD19	MyHC or other cardiac antigens
Primary target cells	B cells and plasmablasts	B cells and plasmablasts	Cardiomyocytes and cardiac antigen-presenting cells
Mechanism of action	Depletion of B cells and plasmablasts to reduce autoantibody production	Suppression of autoantibody production by regulating B cell and plasmablast activity	Heart-specific suppression of inflammation
Potential limitations	Not all patients with SLE may respond Less effective against CD19^−^ plasma cells Increased infection risk	Not all patients with SLE may respond Less effective against CD19^−^ plasma cells Increased infection risk	Effect limited to cardiac inflammation May not address systemic lupus Increased infection risk
Ideal usage	B cell-driven SLE	B cell-driven SLE	Lupus myocarditis or heart-specific inflammation
Development stage	Clinically tested	Tested in humanized mice	Under development
References	^131–134^	^136^	–

Another promising strategy for reducing autoreactive inflammation in autoimmune diseases is CAR-T_reg_ cells, which involves antigen-specific T_reg_ cells designed for enhanced targeting and immunoregulatory function. In a recent study, CAR-T_reg_ cells specific for the myelin oligodendrocyte glycoprotein peptides, a well-established antigen in multiple sclerosis, showed successful localization in the central nervous system, effective suppression of pathogenic T_eff_ cells and delayed disease onset in mice with experimental autoimmune encephalomyelitis^135^. For SLE treatment, human CAR-T_reg_ cells targeting the B cell antigen CD19 (Fox19CAR-T_reg_) were developed and adoptively transferred into humanized mice^136^ (Table 2). This CAR-T_reg_ cell therapy effectively resolved inflammation in the lungs and kidneys through the reduction of circulating B cells and autoantibody production^136^. Given these findings, CAR-T_reg_ cells targeting MyHC or other cardiac antigens could represent a promising therapeutic strategy for lupus myocarditis treatment (Table 2). The cardiac antigen-specific CAR-T_reg_ cell approach would provide selective immunoregulation within the heart, protecting against lupus myocarditis. It can also potentially benefit other autoimmune cardiac disorders, not limited to B cell-driven pathology. However, cardiac antigen-specific CAR-T_reg_ therapies would not address SLE symptoms in other organs and are at an early conceptual stage.

## Conclusion

SLE is a heterogeneous autoimmune disease that can affect single or multiple organs simultaneously, posing significant challenges for diagnosis and treatment^137^. Despite extensive research identifying diverse cellular and molecular pathways contributing to SLE, tissue-specific autoantigens or disease mechanisms remain largely unknown^138–140^. The hallmark lupus autoantibodies play a critical role in SLE pathogenesis^6^. However, they primarily target nuclear antigens ubiquitous across organs and cells without tissue specificity, indicating the limitation in their diagnostic and therapeutic utility.

Myocarditis is one of the most severe cardiovascular complications of SLE^4^. Although the use of corticosteroids has significantly reduced the incidence of lupus myocarditis since 1975, the mortality rate remains remarkably high, between 10% and 23%^4^. Mechanistically, hallmark SLE antibodies and T cells may contribute to lupus myocarditis by recognizing nuclear antigens exposed by initial cardiac damage or cross-reactive targets in the heart. However, not all patients with lupus myocarditis test positive for these autoantibodies, and studies report no differences in their titers between patients with SLE with and without myocarditis, suggesting additional pathogenic mechanisms remain undiscovered^35–38^.

Autoimmunity against MyHC has long been investigated as a key mechanism driving autoimmune myocarditis^19^. The recent emergence of ICI-associated myocarditis has further highlighted the pathogenic role of MyHC-specific autoreactive T cells in autoimmune myocarditis across both clinical and preclinical settings^17,18^. However, the role of adaptive immunity targeting MyHC and other cardiac antigens in lupus myocarditis remains understudied. In this Review, we propose a two-stage model for lupus myocarditis pathogenesis: (1) an initial phase driven by anti-nucleoprotein antibodies and T cells and (2) a secondary phase involving autoimmunity against both nuclear and cardiac antigens (Fig. 2). However, evidence is currently insufficient to validate this model. To advance our understanding of lupus myocarditis, robust investigations incorporating both clinical and animal studies are required.

Identifying heart-specific antigens, along with their corresponding autoreactive T cells and autoantibodies, may provide a novel avenue for diagnosis and treatment of lupus myocarditis. MyHC or other heart antigens already identified could serve as promising therapeutic targets. Advanced molecular techniques such as single-cell RNA sequencing, spatial transcriptomics and proteomics hold promise for better elucidating the complex mechanisms underlying lupus myocarditis.

## References

[1] Tian, J. et al. Global epidemiology of systemic lupus erythematosus: a comprehensive systematic analysis and modelling study. *Ann. Rheum. Dis.* **82**, 351–356 (2023). 36241363 10.1136/ard-2022-223035 PMC9933169

[2] Gomez-Banuelos, E., Fava, A. & Andrade, F. An update on autoantibodies in systemic lupus erythematosus. *Curr. Opin. Rheumatol.* **35**, 61–67 (2023). 36695053 10.1097/BOR.0000000000000922 PMC9881844

[3] Abu-Shakra, M. et al. Mortality studies in systemic lupus erythematosus. Results from a single center. II. Predictor variables for mortality. *J. Rheumatol.* **22**, 1265–1270 (1995). 7562756

[4] Guglin, M., Smith, C. & Rao, R. The spectrum of lupus myocarditis: from asymptomatic forms to cardiogenic shock. *Heart Fail. Rev.* **26**, 553–560 (2021). 33210224 10.1007/s10741-020-10054-w

[5] du Toit, R. et al. Lupus myocarditis: review of current diagnostic modalities and their application in clinical practice. *Rheumatology* **62**, 523–534 (2023). 35861382 10.1093/rheumatology/keac409

[6] Pisetsky, D. S. & Lipsky, P. E. New insights into the role of antinuclear antibodies in systemic lupus erythematosus. *Nat. Rev. Rheumatol.* **16**, 565–579 (2020). 32884126 10.1038/s41584-020-0480-7 PMC8456518

[7] Dema, B. & Charles, N. Autoantibodies in SLE: specificities, isotypes and receptors. *Antibodies* **5**, 2 (2016). 31557984 10.3390/antib5010002 PMC6698872

[8] Gartshteyn, Y. et al. Endomyocardial biopsies in the diagnosis of myocardial involvement in systemic lupus erythematosus. *Lupus* **29**, 199–204 (2020). 31924147 10.1177/0961203319897116 PMC7261237

[9] Lee, S. Y. et al. Fatal myopericarditis in a patient with lupus erythematosus supported by extracorporeal membrane oxygenation: a case report. *J. Rheum. Dis.* **28**, 165–170 (2021). 37475997 10.4078/jrd.2021.28.3.165 PMC10324896

[10] Abdirama, D. et al. Nuclear antigen-reactive CD4^+^ T cells expand in active systemic lupus erythematosus, produce effector cytokines, and invade the kidneys. *Kidney Int.* **99**, 238–246 (2021). p. 32592813 10.1016/j.kint.2020.05.051

[11] Riemekasten, G. et al. T cell reactivity against the SmD1(83–119) C terminal peptide in patients with systemic lupus erythematosus. *Ann. Rheum. Dis.* **61**, 779–785 (2002). 12176801 10.1136/ard.61.9.779 PMC1754211

[12] Kattah, N. H. et al. Tetramers reveal IL-17-secreting CD4^+^ T cells that are specific for U1-70 in lupus and mixed connective tissue disease. *Proc. Natl Acad. Sci. USA* **112**, 3044–3049 (2015). 25713364 10.1073/pnas.1424796112 PMC4364210

[13] Psarras, A., Wittmann, M. & Vital, E. M. Emerging concepts of type I interferons in SLE pathogenesis and therapy. *Nat. Rev. Rheumatol.* **18**, 575–590 (2022). 36097207 10.1038/s41584-022-00826-z

[14] Coss, S. L. et al. The complement system and human autoimmune diseases. *J. Autoimmun.* **137**, 102979 (2023). 36535812 10.1016/j.jaut.2022.102979 PMC10276174

[15] Lv, H. et al. Impaired thymic tolerance to alpha-myosin directs autoimmunity to the heart in mice and humans. *J. Clin. Invest* **121**, 1561–1573 (2011). 21436590 10.1172/JCI44583 PMC3069776

[16] Goldman, J. H. et al. Autoimmunity to alpha myosin in a subset of patients with idiopathic dilated cardiomyopathy. *Br. Heart J.* **74**, 598–603 (1995). 8541162 10.1136/hrt.74.6.598 PMC484112

[17] Axelrod, M. L. et al. T cells specific for alpha-myosin drive immunotherapy-related myocarditis. *Nature* **611**, 818–826 (2022). p. 36385524 10.1038/s41586-022-05432-3 PMC9930174

[18] Won, T. et al. Cardiac myosin-specific autoimmune T cells contribute to immune-checkpoint-inhibitor-associated myocarditis. *Cell Rep.* **41**, 111611 (2022). 36351411 10.1016/j.celrep.2022.111611 PMC11108585

[19] Won, T. et al. Autoimmune myocarditis, old dogs and new tricks. *Circ. Res* **134**, 1767–1790 (2024). 38843292 10.1161/CIRCRESAHA.124.323816

[20] Ryabkova, V. A. et al. Lethal immunoglobulins: autoantibodies and sudden cardiac death. *Autoimmun. Rev.* **18**, 415–425 (2019). 30772491 10.1016/j.autrev.2018.12.005

[21] Lucas, J. A. et al. Programmed death ligand 1 regulates a critical checkpoint for autoimmune myocarditis and pneumonitis in MRL mice. *J. Immunol.* **181**, 2513–2521 (2008). 18684942 10.4049/jimmunol.181.4.2513 PMC2587295

[22] Wang, J. et al. PD-1 deficiency results in the development of fatal myocarditis in MRL mice. *Int Immunol.* **22**, 443–452 (2010). 20410257 10.1093/intimm/dxq026

[23] Gall, A. et al. Autoimmunity initiates in nonhematopoietic cells and progresses via lymphocytes in an interferon-dependent autoimmune disease. *Immunity* **36**, 120–131 (2012). 22284419 10.1016/j.immuni.2011.11.018 PMC3269499

[24] Hasham, M. G. et al. Systemic autoimmunity induced by the TLR7/8 agonist Resiquimod causes myocarditis and dilated cardiomyopathy in a new mouse model of autoimmune heart disease. *Dis. Model Mech.* **10**, 259–270 (2017). 28250051 10.1242/dmm.027409 PMC5374321

[25] Caforio, A. L. P. et al. Diagnosis and management of myocardial involvement in systemic immune-mediated diseases: a position statement of the European Society of Cardiology Working Group on Myocardial and Pericardial Disease. *Eur. Heart J.* **38**, 2649–2662 (2017). 28655210 10.1093/eurheartj/ehx321

[26] Seferovic, P. M. et al. Heart Failure Association of the ESC, Heart Failure Society of America and Japanese Heart Failure Society Position statement on endomyocardial biopsy. *Eur. J. Heart Fail.* **23**, 854–871 (2021). 34010472 10.1002/ejhf.2190

[27] Pons-Estel, G. J. et al. Predictors of cardiovascular damage in patients with systemic lupus erythematosus: data from LUMINA (LXVIII), a multiethnic US cohort. *Rheumatology* **48**, 817–822 (2009). 19454606 10.1093/rheumatology/kep102 PMC2722811

[28] Schoenfeld, S. R., Kasturi, S. & Costenbader, K. H. The epidemiology of atherosclerotic cardiovascular disease among patients with SLE: a systematic review. *Semin Arthritis Rheum.* **43**, 77–95 (2013). 23422269 10.1016/j.semarthrit.2012.12.002

[29] Luetkens, J. A. et al. Comparison of Original and 2018 Lake Louise Criteria for Diagnosis of Acute Myocarditis: results of a validation cohort. *Radio. Cardiothorac. Imaging* **1**, e190010 (2019). 10.1148/ryct.2019190010 PMC7978026 33778510

[30] Davenport, M. S. et al. Use of Intravenous iodinated contrast media in patients with kidney disease: consensus statements from the American College of Radiology and the National Kidney Foundation. *Radiology* **294**, 660–668 (2020). 31961246 10.1148/radiol.2019192094

[31] Alchammas, J. et al. The evaluation of lupus myocarditis with ^13^N-ammonia and ^18^F-FDG PET. *J. Nucl. Med Technol.* **44**, 210–211 (2016). 26769599 10.2967/jnmt.115.165639

[32] du Toit, R. et al. Myocardial injury in systemic lupus erythematosus according to cardiac magnetic resonance tissue characterization: clinical and echocardiographic features. *Lupus* **29**, 1461–1468 (2020). 32631204 10.1177/0961203320936748

[33] Friedrich, M. G. et al. Cardiovascular magnetic resonance in myocarditis: a JACC White Paper. *J. Am. Coll. Cardiol.* **53**, 1475–1487 (2009). 19389557 10.1016/j.jacc.2009.02.007 PMC2743893

[34] Logar, D. et al. Possible association between anti-Ro antibodies and myocarditis or cardiac conduction defects in adults with systemic lupus erythematosus. *Ann. Rheum. Dis.* **49**, 627–629 (1990). 2396870 10.1136/ard.49.8.627 PMC1004179

[35] Thomas, G. et al. Lupus myocarditis: initial presentation and longterm outcomes in a multicentric series of 29 patients. *J. Rheumatol.* **44**, 24–32 (2017). 28042125 10.3899/jrheum.160493

[36] Ramirez, G. A. et al. Distinctive clinical traits of lupus-related myocarditis: a multicentre retrospective study. *Rheumatology* (Oxford). **64**, 1904−1911 10.1093/rheumatology/keae376 (2025). 10.1093/rheumatology/keae376 PMC11962914 39047157

[37] Zawadowski, G. M. et al. A contemporary case series of lupus myocarditis. *Lupus* **21**, 1378–1384 (2012). 22892209 10.1177/0961203312456752

[38] du Toit, R. et al. Serum cytokine levels associated with myocardial injury in systemic lupus erythematosus. *Rheumatology* **60**, 2010–2021 (2021). 33221897 10.1093/rheumatology/keaa540

[39] Ben-Shabat, N. et al. Anti-Ro and anti-La seropositivity is associated with increased rates of ischemic heart disease in adults: results from a large population-based study. *Atherosclerosis* **396**, 117626 (2024). 39002391 10.1016/j.atherosclerosis.2024.117626

[40] Lazzerini, P. E. et al. Anti-Ro/SSA-associated corrected QT interval prolongation in adults: the role of antibody level and specificity. *Arthritis Care Res.* **63**, 1463–1470 (2011). 10.1002/acr.20540 21739618

[41] Xiao, G. Q., Hu, K. & Boutjdir, M. Direct inhibition of expressed cardiac L- and T-type calcium channels by igg from mothers whose children have congenital heart block. *Circulation* **103**, 1599–1604 (2001). 11257091 10.1161/01.cir.103.11.1599

[42] Buyon, J. P. & Winchester, R. Congenital complete heart block. A human model of passively acquired autoimmune injury. *Arthritis Rheum.* **33**, 609–614 (1990). 2346516 10.1002/art.1780330502

[43] Eloranta, M. L. et al. A possible mechanism for endogenous activation of the type I interferon system in myositis patients with anti-Jo-1 or anti-Ro 52/anti-Ro 60 autoantibodies. *Arthritis Rheum.* **56**, 3112–3124 (2007). 17763410 10.1002/art.22860

[44] Miranda-Carus, M. E. et al. Anti-SSA/Ro and anti-SSB/La autoantibodies bind the surface of apoptotic fetal cardiocytes and promote secretion of TNF-alpha by macrophages. *J. Immunol.* **165**, 5345–5351 (2000). 11046070 10.4049/jimmunol.165.9.5345

[45] Ambrosi, A., Sonesson, S. E. & Wahren-Herlenius, M. Molecular mechanisms of congenital heart block. *Exp. Cell Res.* **325**, 2–9 (2014). 24434353 10.1016/j.yexcr.2014.01.003

[46] Knight, J. S., Branch, D. W. & Ortel, T. L. Antiphospholipid syndrome: advances in diagnosis, pathogenesis, and management. *BMJ* **380**, e069717 (2023). 36849186 10.1136/bmj-2021-069717

[47] Elshikha, A. S. et al. TLR7 activation accelerates cardiovascular pathology in a mouse model of lupus. *Front Immunol.* **13**, 914468 (2022). 35860280 10.3389/fimmu.2022.914468 PMC9289616

[48] Myhr, K. A. et al. Myocardial fibrosis associates with lupus anticoagulant in patients with systemic lupus erythematosus. *Int. J. Cardiovasc. Imaging* **40**, 127–137 (2024). 37814154 10.1007/s10554-023-02970-3 PMC10774215

[49] Nihoyannopoulos, P. et al. Cardiac abnormalities in systemic lupus erythematosus. Association with raised anticardiolipin antibodies. *Circulation* **82**, 369–375 (1990). 2372888 10.1161/01.cir.82.2.369

[50] Farina, N. et al. Antiphospholipid antibody positivity in early systemic lupus erythematosus is associated with subsequent vascular events. *Rheumatology* **62**, 2252–2256 (2023). 36227113 10.1093/rheumatology/keac596 PMC10234200

[51] Nevras, V. et al. Acute coronary syndromes in antiphospholipid syndrome-above suspicion: a systematic review. *Curr. Probl. Cardiol.* **48**, 101503 (2023). 36402221 10.1016/j.cpcardiol.2022.101503

[52] Marnach, M., VanWinter, J. & Watson, W. Myocarditis: an unusual cause of postpartum fever in pregnancy complicated by antiphospholipid syndrome. *Am. J. Perinatol.* **24**, 405–408 (2007). 17624816 10.1055/s-2007-984406

[53] Lai, A. C. et al. A case report of acute heart failure and cardiogenic shock caused by catastrophic antiphospholipid syndrome and lupus myocarditis. *Eur. Heart J. Case Rep.* **6**, ytac446 (2022). 36504504 10.1093/ehjcr/ytac446 PMC9728516

[54] De Scheerder, I. K. et al. Humoral immune response against contractile proteins (actin and myosin) during cardiovascular disease. *Eur. Heart J.* **12**, 88–94 (1991). 1915462 10.1093/eurheartj/12.suppl_d.88

[55] Neumann, D. A. et al. Circulating heart-reactive antibodies in patients with myocarditis or cardiomyopathy. *J. Am. Coll. Cardiol.* **16**, 839–846 (1990). 2229805 10.1016/s0735-1097(10)80331-6

[56] Lauer, B. et al. Antimyosin autoantibodies are associated with deterioration of systolic and diastolic left ventricular function in patients with chronic myocarditis. *J. Am. Coll. Cardiol.* **35**, 11–18 (2000). 10636253 10.1016/s0735-1097(99)00485-4

[57] Nussinovitch, U. & Shoenfeld, Y. The clinical and diagnostic significance of anti-myosin autoantibodies in cardiac disease. *Clin. Rev. Allergy Immunol.* **44**, 98–108 (2013). 21207194 10.1007/s12016-010-8229-8

[58] Wolf, R. E., King, J. W. & Brown, T. A. Antimyosin antibodies and constrictive pericarditis in lupus erythematosus. *J. Rheumatol.* **15**, 1284–1287 (1988). 3054097

[59] Fleischer, S. et al. An engineered human cardiac tissue model reveals contributions of systemic lupus erythematosus autoantibodies to myocardial injury. *Nat. Cardiovasc. Res.* **3**, 1123–1139 (2024). 39195859 10.1038/s44161-024-00525-w PMC11399098

[60] Suarez-Fueyo, A., Bradley, S. J. & Tsokos, G. C. T cells in systemic lupus erythematosus. *Curr. Opin. Immunol.* **43**, 32–38 (2016). 27636649 10.1016/j.coi.2016.09.001 PMC5125867

[61] Sharabi, A. & Tsokos, G. C. T cell metabolism: new insights in systemic lupus erythematosus pathogenesis and therapy. *Nat. Rev. Rheumatol.* **16**, 100–112 (2020). 31949287 10.1038/s41584-019-0356-x

[62] Perl, A. Oxidative stress in the pathology and treatment of systemic lupus erythematosus. *Nat. Rev. Rheumatol.* **9**, 674–686 (2013). 24100461 10.1038/nrrheum.2013.147 PMC4046645

[63] Enyedy, E. J. et al. Fc epsilon receptor type I gamma chain replaces the deficient T cell receptor zeta chain in T cells of patients with systemic lupus erythematosus. *Arthritis Rheum.* **44**, 1114–1121 (2001). 11352243 10.1002/1529-0131(200105)44:5<1114::AID-ANR192>3.0.CO;2-B

[64] Richardson, B. et al. Evidence for impaired T cell DNA methylation in systemic lupus erythematosus and rheumatoid arthritis. *Arthritis Rheum.* **33**, 1665–1673 (1990). 2242063 10.1002/art.1780331109

[65] Krishnan, S. et al. Alterations in lipid raft composition and dynamics contribute to abnormal T cell responses in systemic lupus erythematosus. *J. Immunol.* **172**, 7821–7831 (2004). 15187166 10.4049/jimmunol.172.12.7821

[66] Fernandez, D. R. et al. Activation of mammalian target of rapamycin controls the loss of TCRzeta in lupus T cells through HRES-1/Rab4-regulated lysosomal degradation. *J. Immunol.* **182**, 2063–2073 (2009). 19201859 10.4049/jimmunol.0803600 PMC2676112

[67] Koga, T. et al. Promotion of calcium/calmodulin-dependent protein kinase 4 by GLUT1-dependent glycolysis in systemic lupus erythematosus. *Arthritis Rheumatol.* **71**, 766–772 (2019). 30462889 10.1002/art.40785

[68] Monneaux, F. et al. Murine models of systemic lupus erythematosus: B and T cell responses to spliceosomal ribonucleoproteins in MRL/Fas(lpr) and (NZB x NZW)F(1) lupus mice. *Int. Immunol.* **13**, 1155–1163 (2001). 11526096 10.1093/intimm/13.9.1155

[69] Kaliyaperumal, A. et al. Nucleosomal peptide epitopes for nephritis-inducing T helper cells of murine lupus. *J. Exp. Med.* **183**, 2459–2469 (1996). 8676066 10.1084/jem.183.6.2459 PMC2192594

[70] Reynolds, P. et al. Hierarchical self-tolerance to T cell determinants within the ubiquitous nuclear self-antigen La (SS-B) permits induction of systemic autoimmunity in normal mice. *J. Exp. Med.* **184**, 1857–1870 (1996). 8920873 10.1084/jem.184.5.1857 PMC2192903

[71] Tesch, S. et al. Identification and characterization of antigen-specific CD4^+^ T cells targeting renally expressed antigens in human lupus nephritis with two independent methods. *Sci. Rep.* **10**, 21312 (2020). 33277543 10.1038/s41598-020-78223-3 PMC7718878

[72] Gunnarsson, R. et al. Mixed connective tissue disease. *Best. Pr. Res Clin. Rheumatol.* **30**, 95–111 (2016). 10.1016/j.berh.2016.03.002 27421219

[73] Venables, P. J. Mixed connective tissue disease. *Lupus* **15**, 132–137 (2006). 16634365 10.1191/0961203306lu2283rr

[74] O’Brien, R. M. et al. T-cell epitopes on the 70-kDa protein of the (U1)RNP complex in autoimmune rheumatologic disorders. *J. Autoimmun.* **3**, 747–757 (1990). 1708263 10.1016/s0896-8411(05)80041-1

[75] Figliozzi, S. et al. Biopsy-proven lymphocytic myocarditis with heart failure in a middle-aged female patient with mixed connective tissue disease. *JACC Case Rep.* **1**, 171–174 (2019). 34316778 10.1016/j.jaccas.2019.05.032 PMC8301497

[76] Hamana, T. et al. Fulminant myocarditis in a young woman with mixed connective tissue disease: a case report. *Eur. Heart J. Case Rep.* **7**, ytad174 (2023). 37096149 10.1093/ehjcr/ytad174 PMC10122413

[77] Myers, J. M. et al. Cardiac myosin-Th17 responses promote heart failure in human myocarditis. *JCI Insight* **1**, e85851 (2016). 27366791 10.1172/jci.insight.85851 PMC4924810

[78] Pummerer, C. L. et al. Identification of cardiac myosin peptides capable of inducing autoimmune myocarditis in BALB/c mice. *J. Clin. Invest* **97**, 2057–2062 (1996). 8621795 10.1172/JCI118642 PMC507280

[79] Donermeyer, D. L. et al. Myocarditis-inducing epitope of myosin binds constitutively and stably to I-Ak on antigen-presenting cells in the heart. *J. Exp. Med.* **182**, 1291–1300 (1995). 7595200 10.1084/jem.182.5.1291 PMC2192215

[80] Kodama, M. et al. A novel experimental model of giant cell myocarditis induced in rats by immunization with cardiac myosin fraction. *Clin. Immunol. Immunopathol.* **57**, 250–262 (1990). 2208806 10.1016/0090-1229(90)90039-s

[81] Schenkel, J. M. & Masopust, D. Tissue-resident memory T cells. *Immunity* **41**, 886–897 (2014). 25526304 10.1016/j.immuni.2014.12.007 PMC4276131

[82] Frieser, D. et al. Tissue-resident CD8^+^ T cells drive compartmentalized and chronic autoimmune damage against CNS neurons. *Sci. Transl. Med.* **14**, eabl6157 (2022). 35417189 10.1126/scitranslmed.abl6157

[83] Povoleri, G. A. M. et al. Psoriatic and rheumatoid arthritis joints differ in the composition of CD8^+^ tissue-resident memory T cell subsets. *Cell Rep.* **42**, 112514 (2023). 37195862 10.1016/j.celrep.2023.112514 PMC10790246

[84] Bishu, S. et al. CD4^+^ tissue-resident memory t cells expand and are a major source of mucosal tumour necrosis factor alpha in active Crohn’s disease. *J. Crohns Colitis* **13**, 905–915 (2019). 30715262 10.1093/ecco-jcc/jjz010 PMC6939878

[85] Gu, H. J. et al. Expression pattern of tissue-resident memory T cells in cutaneous lupus erythematosus. *Lupus* **30**, 1427–1437 (2021). 34013817 10.1177/09612033211017218

[86] Zhou, M. et al. JAK/STAT signaling controls the fate of CD8^+^CD103^+^ tissue-resident memory T cell in lupus nephritis. *J. Autoimmun.* **109**, 102424 (2020). 32085893 10.1016/j.jaut.2020.102424

[87] Lai, Y. et al. Novel approach to alleviate lupus nephritis: targeting the NLRP3 inflammasome in CD8^+^CD69^+^CD103^+^ T(RM) cells. *J. Transl. Med.* **22**, 1139 (2024). 39716284 10.1186/s12967-024-05951-9 PMC11668076

[88] Tilstra, J. S. et al. Kidney-infiltrating T cells in murine lupus nephritis are metabolically and functionally exhausted. *J. Clin. Invest.* **128**, 4884–4897 (2018). 30130253 10.1172/JCI120859 PMC6205402

[89] Kalinoski, H. et al. Injury-induced myosin-specific tissue-resident memory T cells drive immune checkpoint inhibitor myocarditis. *Proc. Natl Acad. Sci. USA* **121**, e2323052121 (2024). 39378095 10.1073/pnas.2323052121 PMC11494310

[90] Gough, S. C. & Simmonds, M. J. The HLA region and autoimmune disease: associations and mechanisms of action. *Curr. Genom.* **8**, 453–465 (2007). 10.2174/138920207783591690 PMC2647156 19412418

[91] Endreffy, E. et al. HLA class II allele polymorphism in Hungarian patients with systemic lupus erythematosus. *Ann. Rheum. Dis.* **62**, 1017–1018 (2003). 12972487 10.1136/ard.62.10.1017 PMC1754336

[92] Selvaraja, M. et al. Human leucocyte antigens profiling in Malay female patients with systemic lupus erythematosus: are we the same or different?. *Lupus Sci. Med.* **9**, e000554 (2022). 35105721 10.1136/lupus-2021-000554 PMC8808435

[93] Cortes, L. M. et al. HLA class II haplotypes in Mexican systemic lupus erythematosus patients. *Hum. Immunol.* **65**, 1469–1476 (2004). 15603875 10.1016/j.humimm.2004.09.008

[94] Elliott, J. F. et al. Autoimmune cardiomyopathy and heart block develop spontaneously in HLA-DQ8 transgenic IAbeta knockout NOD mice. *Proc. Natl Acad. Sci. USA* **100**, 13447–13452 (2003). 14570980 10.1073/pnas.2235552100 PMC263834

[95] Taylor, J. A. et al. A spontaneous model for autoimmune myocarditis using the human MHC molecule HLA-DQ8. *J. Immunol.* **172**, 2651–2658 (2004). 14764740 10.4049/jimmunol.172.4.2651

[96] Lozano, M. D. et al. Human leukocyte antigen class II associations in patients with idiopathic dilated cardiomyopathy. Myocarditis Treatment Trial Investigators. *J. Card. Fail.* **3**, 97–103 (1997). 9220309 10.1016/s1071-9164(97)90041-5

[97] Arnett, F. C. et al. Molecular analysis of major histocompatibility complex alleles associated with the lupus anticoagulant. *J. Clin. Invest.* **87**, 1490–1495 (1991). 1673688 10.1172/JCI115158 PMC295227

[98] Mori, S. et al. Neoself-antigens are the primary target for autoreactive T cells in human lupus. *Cell* **187**, 6071–6087 e20 (2024). 39276775 10.1016/j.cell.2024.08.025

[99] Cresswell, P. Invariant chain structure and MHC class II function. *Cell* **84**, 505–507 (1996). 8598037 10.1016/s0092-8674(00)81025-9

[100] Sagar, S., Liu, P. P. & Cooper, L. T. Jr. Myocarditis. *Lancet* **379**, 738–747 (2012). 22185868 10.1016/S0140-6736(11)60648-X PMC5814111

[101] Vitzthum von Eckstaedt, H. et al. Immune checkpoint inhibitors and lupus erythematosus. *Pharmaceuticals* **17**, 252 10.3390/ph17020252 (2024) 10.3390/ph17020252 PMC10892070 38399467

[102] Abo El-Khair, S. M. et al. Programmed cell death 1 gene polymorphism as a possible risk for systemic lupus erythematosus in Egyptian females. *Lupus* **28**, 1427–1434 (2019). 31551030 10.1177/0961203319878493

[103] Gao, J. et al. Meta-analysis of programmed cell death 1 polymorphisms with systemic lupus erythematosus risk. *Oncotarget* **8**, 36885–36897 (2017). 28415570 10.18632/oncotarget.16378 PMC5482706

[104] Perl, A. Activation of mTOR (mechanistic target of rapamycin) in rheumatic diseases. *Nat. Rev. Rheumatol.* **12**, 169–182 (2016). 26698023 10.1038/nrrheum.2015.172 PMC5314913

[105] Sowell, R. T. & Marzo, A. L. Resident-memory CD8 T cells and mTOR: generation, protection, and clinical importance. *Front Immunol.* **6**, 38 (2015). 25699054 10.3389/fimmu.2015.00038 PMC4318394

[106] Chan, L. S. et al. Epitope spreading: lessons from autoimmune skin diseases. *J. Invest. Dermatol.* **110**, 103–109 (1998). 9457902 10.1046/j.1523-1747.1998.00107.x

[107] Deshmukh, U. S. et al. Epitope spreading within lupus-associated ribonucleoprotein antigens. *Clin. Immunol.* **117**, 112–120 (2005). 16095971 10.1016/j.clim.2005.07.002

[108] Vignali, D. A., Collison, L. W. & Workman, C. J. How regulatory T cells work. *Nat. Rev. Immunol.* **8**, 523–532 (2008). 18566595 10.1038/nri2343 PMC2665249

[109] Lee, J. H. et al. Inverse correlation between CD4^+^ regulatory T-cell population and autoantibody levels in paediatric patients with systemic lupus erythematosus. *Immunology* **117**, 280–286 (2006). 16423064 10.1111/j.1365-2567.2005.02306.x PMC1782210

[110] Huang, J. et al. Imbalance of Th17 cells, T_reg_ cells and associated cytokines in patients with systemic lupus erythematosus: a meta-analysis. *Front. Immunol*. **15**, 1425847 10.3389/fimmu.2024.1425847 (2024) 10.3389/fimmu.2024.1425847 PMC11288813 39086480

[111] Yu, J. et al. Inflammatory factor-mediated miR-155/SOCS1 signaling axis leads to T_reg_ impairment in systemic lupus erythematosus. *Int. Immunopharmacol*. **141**, 113013 10.1016/j.intimp.2024.113013 (2024) 10.1016/j.intimp.2024.113013 39213866

[112] Shan, J., Jin, H. & Xu, Y. T cell metabolism: a new perspective on Th17/T_reg_ cell imbalance in systemic lupus erythematosus. *Front Immunol.* **11**, 1027 (2020). 32528480 10.3389/fimmu.2020.01027 PMC7257669

[113] Kato, H. & Perl, A. Blockade of T_reg_ cell differentiation and function by the interleukin-21–mechanistic target of rapamycin axis via suppression of autophagy in patients with systemic lupus erythematosus. *Arthritis Rheumatol.* **70**, 427–738 (2017). 10.1002/art.40380 PMC5826851 29161463

[114] Martins, S. et al. Reduced numbers of regulatory T cells in chronic heart failure seems not to be restored by cardiac resynchronization therapy. *BMC Cardiovas. Disord.* **23**, 89 10.1186/s12872-023-03109-x (2023). 10.1186/s12872-023-03109-x PMC9933267 36792985

[115] Wei, Y. et al. CD4^+^ CD25^+^ GARP^+^ regulatory T cells display a compromised suppressive function in patients with dilated cardiomyopathy. *Immunology* **151**, 291–303 (2017). 28207945 10.1111/imm.12728 PMC5461097

[116] Yan, L. et al. Inhibition of microRNA-155 ameliorates experimental autoimmune myocarditis by modulating Th17/T_reg_ immune response. *J. Mol. Med.* **94**, 1063–1079 (2016). 27052830 10.1007/s00109-016-1414-3

[117] Lasrado, N. et al. Dissecting the cellular landscape and transcriptome network in viral myocarditis by single-cell RNA sequencing. *iScience* **25**, 103865 (2022). 35243228 10.1016/j.isci.2022.103865 PMC8861636

[118] Ono, M. et al. Control of autoimmune myocarditis and multiorgan inflammation by glucocorticoid-induced TNF receptor family-related protein(high), Foxp3-expressing CD25^+^ and CD25^−^ regulatory T cells. *J. Immunol.* **176**, 4748–4756 (2006). 16585568 10.4049/jimmunol.176.8.4748

[119] Thonhoff, J. et al. Expanded autologous regulatory T-lymphocyte infusions in ALS. *Neurology* **5**, e465 10.1212/NXI.0000000000000465 (2018) 10.1212/NXI.0000000000000465 PMC5961523 29845093

[120] Voskens, C. et al. Autologous regulatory T-­cell transfer in refractory ulcerative colitis with concomitant primary sclerosing cholangitis. *Gut* **72**, 49–53 (2023). 35428657 10.1136/gutjnl-2022-327075 PMC9763232

[121] Scalapino, K. J. et al. Suppression of disease in New Zealand Black/New Zealand White lupus-prone mice by adoptive transfer of ex vivo expanded regulatory T cells. *J. Immunol.* **177**, 1451–1459 (2006). 16849451 10.4049/jimmunol.177.3.1451

[122] Humrich, J. Y. et al. Homeostatic imbalance of regulatory and effector T cells due to IL-2 deprivation amplifies murine lupus. *Proc. Natl Acad. Sci. USA* **107**, 204–209 (2010). 20018660 10.1073/pnas.0903158107 PMC2806746

[123] Cui, C. et al. CD4^+^ T-cell endogenous cystathionine gamma lyase-hydrogen sulfide attenuates hypertension by sulfhydrating liver kinase B1 to promote T regulatory cell differentiation and proliferation. *Circulation* **142**, 1752–1769 (2020). 32900241 10.1161/CIRCULATIONAHA.119.045344

[124] Feng, J. et al. Regulatory T cells ameliorate hyperhomocysteinaemia-accelerated atherosclerosis in *apoE*^*−/−*^ mice. *Cardiovasc. Res.* **84**, 155–163 (2009). 19502284 10.1093/cvr/cvp182

[125] Shi, Y. et al. Regulatory T cells protect mice against coxsackievirus-induced myocarditis through the transforming growth factor beta-coxsackie-adenovirus receptor pathway. *Circulation* **121**, 2624–2634 (2010). 20530002 10.1161/CIRCULATIONAHA.109.893248

[126] Bluestone, J. A. et al. Opportunities for T_reg_ cell therapy for the treatment of human disease. *Front. Immunol.* **14**, 1166135 (2023). 37153574 10.3389/fimmu.2023.1166135 PMC10154599

[127] Desreumaux, P. et al. Safety and efficacy of antigen-specific regulatory T-cell therapy for patients with refractory Crohn’s disease. *Gastroenterology* **143**, 1207–1217 (2012). 22885333 10.1053/j.gastro.2012.07.116

[128] Eggenhuizen, P. J. et al. Smith-specific regulatory T cells halt the progression of lupus nephritis. *Nat. Commun.* **15**, 899 (2024). 38321013 10.1038/s41467-024-45056-x PMC10847119

[129] Larson, R. M. M., Recent advances and discoveries in the mechanisms and functions of CAR T cells. *Nat. Rev. Cancer* **21**, 145–161 (2021). 33483715 10.1038/s41568-020-00323-z PMC8353572

[130] Jackson, H. J., Rafiq, S. & Brentjens, R. J. Driving CAR T-cells forward. *Nat. Rev. Clin. Oncol.* **13**, 370–383 (2016). 27000958 10.1038/nrclinonc.2016.36 PMC5529102

[131] Jin, X. et al. Therapeutic efficacy of anti-CD19 CAR-T cells in a mouse model of systemic lupus erythematosus. *Cell Mol. Immunol.* **18**, 1896–1903 (2021). 32472023 10.1038/s41423-020-0472-1 PMC8322088

[132] Kansal, R. et al. Sustained B cell depletion by CD19-targeted CAR T cells is a highly effective treatment for murine lupus. *Sci. Transl. Med.* **11**, eaav1648 10.1126/scitranslmed.aav1648 (2019) 10.1126/scitranslmed.aav1648 PMC8201923 30842314

[133] Mackensen, A. et al. Anti-CD19 CAR T cell therapy for refractory systemic lupus erythematosus. *Nat. Med.* **28**, 2124–2132 (2022). 36109639 10.1038/s41591-022-02017-5

[134] Muller, F. et al. CD19 CAR T-cell therapy in autoimmune disease—a case series with follow-up. *N. Engl. J. Med.* **390**, 687–700 (2024). 38381673 10.1056/NEJMoa2308917

[135] Frikeche, J. et al. MOG-specific CAR T_regs_: a novel approach to treat multiple sclerosis. *J. Neuroinflammation* **21**, 268 (2024). 39428507 10.1186/s12974-024-03262-w PMC11490997

[136] Doglio, M. et al. Regulatory T cells expressing CD19-targeted chimeric antigen receptor restore homeostasis in systemic lupus erythematosus. *Nat. Commun.* **15**, 2542 (2024) 10.1038/s41467-024-46448-9 PMC10973480 38538608

[137] Allen, M. E., Rus, V. & Szeto, G. L. Leveraging heterogeneity in systemic lupus erythematosus for new therapies. *Trends Mol. Med.* **27**, 152–171 (2021). 33046407 10.1016/j.molmed.2020.09.009 PMC8667782

[138] Chaussabel, D. et al. A modular analysis framework for blood genomics studies: application to systemic lupus erythematosus. *Immunity* **29**, 150–164 (2008). 18631455 10.1016/j.immuni.2008.05.012 PMC2727981

[139] Der, E. et al. Tubular cell and keratinocyte single-cell transcriptomics applied to lupus nephritis reveal type I IFN and fibrosis relevant pathways. *Nat. Immunol.* **20**, 915–927 (2019). 31110316 10.1038/s41590-019-0386-1 PMC6584054

[140] Banchereau, R. et al. Personalized immunomonitoring uncovers molecular networks that stratify lupus patients. *Cell* **165**, 551–565 (2016). 27040498 10.1016/j.cell.2016.03.008 PMC5426482

[141] Young, N. A. et al. Pathological manifestation of autoimmune myocarditis is detected prior to glomerulonephritis in a murine model of lupus nephritis. *Lupus* **29**, 1790–1799 (2020). 33045900 10.1177/0961203320948959 PMC7641903

